# Fine mapping of *QYrsv.swust-*1BL for resistance to stripe rust in durum wheat Svevo

**DOI:** 10.3389/fpls.2024.1395223

**Published:** 2024-06-12

**Authors:** Xinli Zhou, Guoyun Jia, Yuqi Luo, Xin Li, Lin Cai, Xianming Chen, Zhensheng Kang

**Affiliations:** ^1^ Wheat Research Institute, School of Life Sciences and Engineering, Southwest University of Science and Technology, Mianyang, Sichuan, China; ^2^ College of Tobacco Science of Guizhou University, Key Laboratory of Plant Resource Conservation and Germplasm Innovation in Mountainous Region (Ministry of Education), Guizhou Key Lab of Agro-Bioengineering, Guiyang, China; ^3^ US Department of Agriculture, Agricultural Research Service, Wheat Health, Genetics, and Quality Research Unit, and Department of Plant Pathology, Washington State University, Pullman, WA, United States; ^4^ State Key Laboratory of Crop Stress Biology in Arid Areas and College of Plant Protection, Northwest A&F University, Xianyang, Shaanxi, China

**Keywords:** stripe rust, resistance gene, durum wheat, fine mapping, quantitative trait locus

## Abstract

Stripe rust, caused by *Puccinia striiformis* f. sp. *tritici* (*Pst*), is a serious disease that affects wheat worldwide. There is a great need to develop cultivars with combinations of all-stage resistance (ASR) and adult-plant resistance (APR) genes for sustainable control of the disease. *QYrsv.swust-1BL* in the Italian durum wheat (*Triticum turgidum* ssp. *durum*) cultivar Svevo is effective against *Pst* races in China and Israel, and the gene has been previously mapped to the long arm of chromosome 1B. The gene is flanked by SNP (single nucleotide polymorphism) markers *IWB5732* and *IWB4839* (0.75 cM). In the present study, we used high-density 660K SNP array genotyping and the phenotypes of 137 recombinant inbred lines (RILs) to fine map the *QYrsv.swust-1BL* locus within a 1.066 Mb region in durum wheat Svevo (RefSeq Rel. 1.0) on chromosome arm 1BL. The identified 1.066 Mb region overlaps with a previously described map of *Yr29/QYr.ucw-1BL*, a stripe rust APR gene. Twenty-five candidate genes for *QYrsv.swut-1BL* were identified through comparing polymorphic genes within the 1.066 Mb region in the resistant cultivar. SNP markers were selected and converted to Kompetitive allele-specific polymerase chain reaction (KASP) markers. Five KASP markers based on SNP were validated in a F_2_ and F_2:3_ breeding population, providing further compelling evidence for the significant effects of *QYrsv.swut-1BL*. These markers should be useful in marker-assisted selection for incorporating *Yr29/QYrsv.swust-1BL* into new durum and common wheat cultivars for resistance to stripe rust.

## Introduction

Wheat is a widely cultivated grain crop and is critical for global food security. Thus, it is important to limit pathogen-caused losses in wheat yield. Stripe rust (Yellow rust, *Yr*), caused by the fungal pathogen *Puccinia striiformis* f. sp. *tritici* (*Pst*), is a growing threat to global wheat production (Chen, 2005, 2020). Although fungicides can control stripe rust, this approach can be costly and harmful to humans, animals, and the environment. The most economical, effective, and environmentally friendly approach to controlling stripe rust is to develop resistant cultivars. However, because of co-evolution pressures, race-specific resistance can be ultimately overcome by new races of the pathogen, often within just a few years (Beddow et al., 2015). There is an urgent need to identify effective stripe rust-resistant genes and develop new resistant cultivars.

At present, 86 formally named resistance genes, more than 70 temporarily named genes, and more than 380 quantitative trait loci (QTL) for resistance to stripe rust have been identified from common wheat, durum wheat, and wild relatives (Bansal et al., 2014; Wang and Chen, 2017; Feng et al., 2018; Nsabiyera et al., 2018; Gessese et al., 2019; Maccaferri et al., 2019; Pakeerathan et al., 2019; Li et al., 2020; Singh et al., 2021; Klymiuk et al., 2022; Yao et al., 2022; Feng et al., 2023; Zhu et al., 2023). However, most of the genes were overcome by the pathogen shortly after being introduced to commercial cultivars. Race-specific (mostly all-stage resistance genes) and non-race-specific [mostly adult-plant resistance (APR) genes] are two major classes of resistance to wheat stripe rust (Chen et al., 2013). Race-specific resistance genes typically encode nucleotide-binding site-leucine-rich repeat (NBS-LRR) resistance proteins. These proteins can detect the presence of pathogen effectors or modify the defense proteins they produce in the host, thereby triggering hypersensitive reactions and reducing the growth of pathogens (Periyannan et al., 2013; Zhang et al., 2017; Chen et al., 2018; Marchal et al., 2018). However, many race-specific resistance genes in commercial cultivars are no longer effective. This is because amino acid changes in the effectors can help the pathogen evade recognition by the nucleotide-binding leucine-rich repeat (NB-LRR) genes (Chen et al., 2017; Salcedo et al., 2017; Cobo et al., 2019). By contrast, APR genes typically show more durable but less strong resistance than all-stage race-specific genes (Krattinger et al., 2009; Singh et al., 2011; Chen et al., 2013). Three APR genes against wheat stripe rust have been cloned, encoding proteins that are more complex and diverse than NBS-LRR. *Yr18* encodes an ATP-binding cassette (ABC) transporter (Krattinger et al., 2009); *Yr36* encodes a kinase and a putative START lipid-binding domain (Fu et al., 2009; Gou et al., 2015); and *Yr46* encodes a hexose transporter (Moore et al., 2015). Using APR is a demonstrated approach to developing wheat cultivars with durable resistance to stripe rust (Chen et al., 2013; Liu et al., 2018, 2019).

Stripe rust resistance QTL *QYrsv.swust-1BL* was previously mapped between the linked SNP markers *IWB5732* and *IWB4839* in a 0.75 cM (1.74 Mb) region derived from durum wheat cultivar Svevo (Zhou et al., 2021), which overlaps the map of *Yr29*, a long-term utilized gene for APR to stripe rust (William et al., 2003; Kolmer, 2015). The known pleiotropic APR gene *Yr29/Lr46/Sr58/Pm39* has been reported in several studies (Ren et al., 2017; Cobo et al., 2018, 2019; Herrera-Foessel et al., 2011; Ponce-Molina et al., 2018; Li et al., 2020). *Yr29/Lr46* (Singh et al., 1998; William et al., 2003) provides partial APR to rust diseases (Herrera-Foessel et al., 2015) and powdery mildew (Lillemo et al., 2008, 2013) in wheat. *Yr29/Lr46* is also associated with the leaf tip necrosis (*Ltn*) gene *Ltn2* (William et al., 2003; Rosewarne et al., 2006). *QYrsv.swust*-*1BL* explained 11.0% to 34.4% of the phenotypic variation and was effective across all tested environments in China and Israel. The phenotypic variance explained by *QYrsv.swust*-*1BL* and *Yr29* was very similar (Zhou et al., 2021). However, it is not certain whether the two genes are the same gene, allelic genes, or closely linked genes. In addition, the flanking markers were not sufficiently close for efficient marker-assisted selection.

The objectives of this study were to genotype recombinant inbred lines (RILs) used in the previous study (Zhou et al., 2021) using the wheat 660K SNP iSelect array, produce a high-resolution map for the resistance gene *QYrsv.swust*-*1BL* in Svevo with more markers, identify candidates for this resistance gene, transfer *QYrsv.swust*-*1BL* into common wheat, and develop user-friendly markers for validation to hasten the use of this gene in breeding programs.

## Materials and methods

### Plant materials

To fine-map the locus of *QYrsv.swust*-*1BL* for stripe rust APR in Svevo, 137 RILs developed from Svevo/Zavitan (Avni et al., 2014; Zhou et al., 2021) were used as a mapping population. To transfer and validate stripe rust resistance from durum wheat Svevo to the Chinese common wheat (*T. aestivum*) background, Svevo was crossed with Mianmai 37 (MM 37), a widely grown common wheat cultivar developed by the Wheat Research Institute, Mianyang Academy of Agricultural Sciences, Sichuan, China. However, this cultivar has become susceptible to stripe rust in recent years. Twelve F_1_ plants derived from MM 37/Svevo were selfed to generate 474 F_2_ plants. Due to sterility, only 318 F_2:3_ families were successfully harvested from 474 F_2_ plants. F_2_ plants correspond to 318 F_2:3_ lines, which were phenotyped for stripe rust response and used to detect the availability of markers. Common wheat cultivars Jinmai 47 (JM 47) and Mingxian 169 (MX 169) were used in the phenotyping experiments as stripe rust-susceptible checks.

### Field evaluation

In 2019 and 2020, the 137 RILs of Svevo/Zavitan were phenotyped for their responses to stripe rust in Yangling (YL, 34.292N, 108.077E), Shaanxi Province, and Mianyang (MY, 31.682N, 104.663E), Sichuan Province, China. A randomized complete block design was used, with two replicates in each year at each location. For each line in each replicate, 25 to 30 seeds were sown in a 1-m row, with 25 cm separating the rows. As susceptible checks, JM 47 was planted in every 20^th^ row and MX 169 around the nursery to increase the pathogen inoculum. The YL site was planted on 6 October 2019 and 10 October 2020, and the MY site on 12 November 2019 and 15 November 2020. In Yangling, when flag leaves emerged [Zadoks growth stage (GS) 40] (Zadoks et al., 1974) in late March, the plants were inoculated with a mixture of equal amounts of urediniospores of *Pst* races CYR32, CYR33, and CYR34, which were predominant races in China. The fields in Mianyang were tested for natural *Pst* infection in these two growing seasons, as this region is one of the major *Pst* inoculum sources in China (Chen et al., 2014). The parents, 12 F_1_, and 474 F_2_ plants of MM 37/Svevo were grown at the research farm of Southwest University of Science and Technology in Mianyang during the 2017–2018 and 2018–2019 growing seasons, respectively. Three hundred eighteen F_2:3_ family seeds were harvested from 474 F_2_ plants and were planted, together with the parental lines, at Mianyang on 12 November 2019 in a randomized complete block design with two replications. Infection type (IT) based on the 0–9 scale (Line and Qayoum, 1992) and disease severity (DS) as percentage of infected foliage were recorded for each parent and RIL when JM 47 and MX 169 had 80% or higher DS at Zadoks growth stages (GS) 65–69.

### Phenotypic analysis

Relative to the phenotypic scores of the parents and susceptible checks, the RILs of Svevo/Zavitan and the F_2:3_ families of MM 37/Svevo were classified into the groups of homozygous resistant (HR) and homozygous susceptible (HS). In addition, the segregating F_2:3_ families were classified as segregating (Seg.). An analysis of variance (ANOVA) was conducted based on the IT and DS data of the populations in the tests of different years and fields (considered as different environments) using the “AOV” tool in QTL IciMapping V4.2 software (Wang, 2009; Meng et al., 2015).

### DNA extraction, KASP marker development, and genotyping the RIL and F_2_ populations

**Figure 1:**
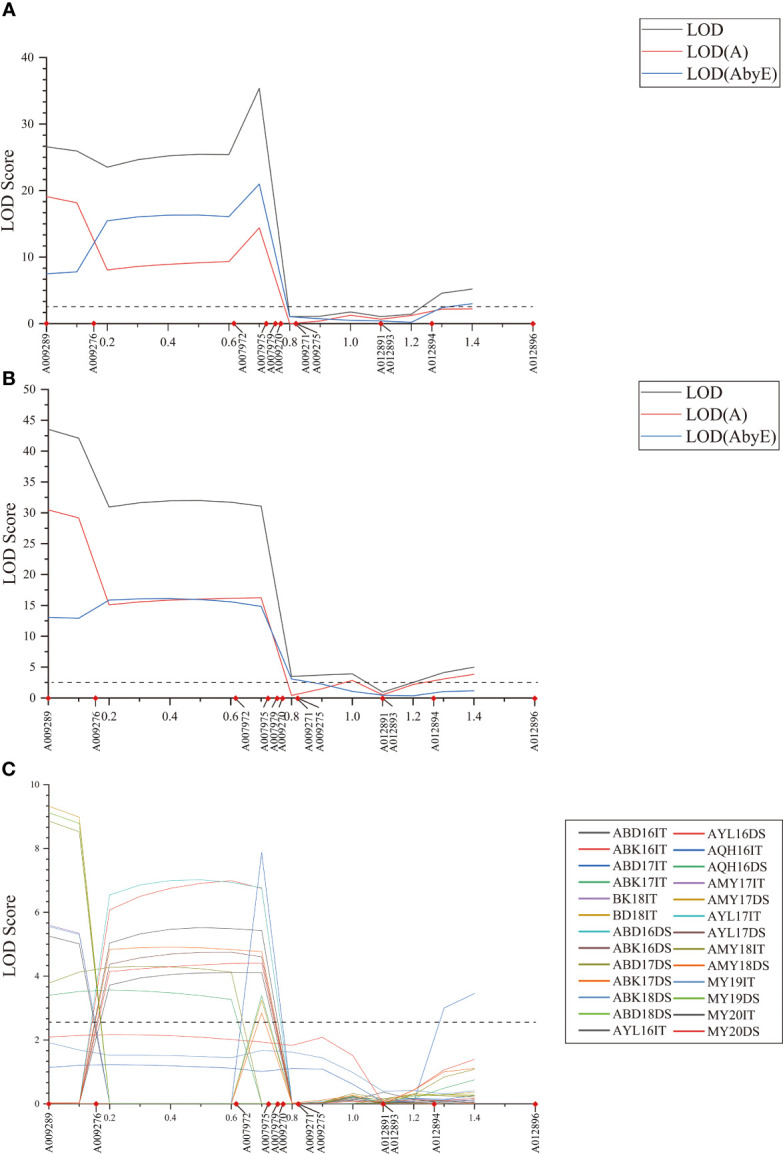
LOD scores of the MET **(A, B)** and BIP **(C)** analyses on infection type (IT) and disease severity (DS) values in stripe rust resistance *QYrsv.swust-1BL*. LOD, the total Lod score; LOD **(A)**, the additive effect component of the LOD; LOD (AbyE), the environmental by additive effect component of the LOD. The X-axis represents the genetic distance of the chromosomes. The Y-axis represents the threshold LOD score.

Total genomic DNA was extracted from the parents, 137 RILs of Svevo/Zavitan, and 318 F_2_ plants from MM 37/Svevo using the cetyltrimethylammonium bromide (CTAB) method (Anderson et al., 1993). To identify closer SNP markers and saturate the targeted QTL region, the genetic maps of the RIL mapping population produced in the previous studies with the 90K SNP array (Avni et al., 2014; Zhou et al., 2021) and the sequences of closely linked polymorphic SNPs were used to BLAST search version 1.0 of the Chinese Spring and Svevo genome sequences from the International Wheat Genome Consortium (IWGSC) and at the GrainGenes Svevo Genome Browser (Durum Wheat Svevo RefSeq Rel. 1.0), respectively. Svevo, Zavitan, and MM 37 were first genotyped with the 660K SNP array by China Golden Marker Biotech Co., Ltd. (Beijing, China). The Kompetitive allele-specific polymerase chain reaction (KASP) markers were developed from the 660K SNP map (http://wheat.pw.usda.gov/ggpages/topics/Wheat660_SNP_array_developed_by_CAAS.pdf). Based on chromosome positions in the 660K SNP map, SNPs within a more conservative target region between *IWB8812* and *IWB4839* were selected for conversion to KASP markers (**Figure 1**). The KASP markers were used to screen the Svevo and Zavitan to confirm polymorphisms before genotyping the entire RIL population. Similarly, before screening the 318 F_2_ plants, MM 37 and Svevo were tested with the KASP markers to confirm the polymorphisms before the markers were used to genotype the 318 F_2_ plants from the MM 37/Svevo cross. The design of KASP primers, PCR amplification, and marker detection were conducted following the PolyMarker method (Ramirez-Gonzalez et al., 2015; Wu et al., 2018).

### Construction of genetic maps and QTL mapping

Genome Studio Polyploid Clustering v1.0 (Illumina, New York, USA) was used for genotype calling and clustering. Markers with >20% of missing data were excluded from further analyses. Marker binning was done using pattern segregation methods (Wang, 2009; Meng et al., 2015). The linkage groups were ordered, and non-binned markers were placed in the groups based on their genome positions. The Chi-squared test was used to detect any segregation distortions. Markers with a *p*-value of < 0.01 in the Chi-squared test were removed prior to generating the genetic map.

Genetic linkage maps of the KASP markers were constructed using the previously described procedures (Zhou et al., 2019; 2021). The likelihood of an odd score of 2.5 was set as the threshold for linkages between loci, and QTL mapping was conducted based on the phenotyping and genotyping data using the software IciMapping V4.2 (Wang, 2009; Meng et al., 2015). Mapping was conducted first using the BIP (biparental population) module and the ICIM-ADD model in the years 2019–2020 and by combining the data from the previous years 2016–2018 from all environments. The MET (multi-environmental trials) module was also employed to identify the QTL loci in the years 2016–2020 in all environments. The methodology used for the BIP and MET analyses closely follows the approach described in Zhou et al. (2021). But the mapping parameters were step=0.1 cM and PIN=0.001 (probability of SNP to be included in the model).

### Identification of candidate genes

The probes of the SNP identified in the QTL region were aligned to the newly released Chinese Spring sequences (IWGSC RefSeq V1.0, annotation V1.1, the International Wheat Genome Consortium (IWGSC), https://www.wheatgenome.org/about/iwgsc-2.0) through BLAST searching. This provided physical positions, reference sequences, annotations, and alignments of the polymorphic SNP markers. The SNP probes were also BLAST searched against the Svevo genome sequences at the GrainGenes Svevo Genome Browser (Durum Wheat Svevo RefSeq Rel. 1.0). Annotated genes in the target region were bioinformatically analyzed using the NCBI (https://bl-ast.ncbi.nlm.nih.gov/Blast.cgi) and Genoscope (https://www.cea.fr/drf/ifr-ancoisjacob/Pages/Departements/Genoscope.aspx) databases and the Web site-based software FGENSH (http://www.softberry.com/berry.phtml?topic=fgenesh&group=he-lp&subgroup=gfind) for their putative functions and possible involvement in plant disease resistance to identify candidates of the stripe rust resistance genes.

## Results

### Developing KASP markers

Using the initial markers *IWB8812* and *IWB4839* from the 90K wheat SNP array as the conservative flanking loci, *QYrsv.swust-1BL* from Svevo was mapped in the 1.855 Mb (physical position: 669,649,666 ~ 671,505,587 bp) region corresponding to the Chinese Spring IWGSC RefSeq v1.0 1BL chromosome, which includes the segments previously reported and considered as *QYr.ucw-1BL/Yr29* (Cobo et al., 2019; Zhou et al., 2021). By comparing the genome sequences of durum wheat Svevo RefSeq Rel. 1.0 (the GrainGenes Svevo Genome Browser) and Chinese Spring IWGSC RefSeq v1.0 (https://urgi.versailles.inra.fr/download/iwgsc/), a nucleic acid database was constructed with the genome sequences of Svevo. The sequences of 630,517 SNP probes from the 660K SNP array were compared to the genome sequences of Svevo by BLASTn. All SNP probes that matched the expected sequences of the 660,683,255 ~ 663,334,719 bp region of the Svevo chromosome 1BL were selected. Similarly, in the Chinese Spring genome, SNPs in the 669,649,666 ~ 671,505,587 bp region were identified. Based on both physical positions of the commonly polymorphic SNP loci on 1BL, a total of 42 SNP markers were selected from the wheat 660K SNP array in the 669,649,666 ~ 671,505,587 bp region, from which 21 KASP markers were successfully developed. After confirming the polymorphisms between the parental lines (Svevo and Zavitan), thirteen KASP markers (*A009289*, *A009276*, *A009272*, *A007975*, *A007979*, *A009270*, *A009271*, *A007972*, *A009275*, *A012891*, *A012893*, *A012894*, and *A012896*) successfully distinguished Svevo and Zavitan (**Table 1**). A total of 47 wheat DNA samples, including the 2 parents and 23 resistant and 22 susceptible RILs from the Svevo/Zavitan cross, were selected to verify these markers, which were then tested on 137 RILs from the Svevo/Zavitan cross. The sequences of the 13 KASP markers are shown in **Table 1**. The SNP sequences associated with *QYrsv.swust-1BL* identified from the wheat 660K SNP array are shown in **Table 2**, which were used to design the KASP primer sequence. When these 13 KASP markers were used to genotype MM 37, Svevo, five markers—*A009289*, *A012891*, *A012893*, *A012894*, and *A012896*—were polymorphic between the parents (MM 37, Svevo) and among the tested F_2_ plants from their cross. Indicating that the markers *A009289*, *A012891*, *A012893*, *A012894*, and *A012896* are useful in marker-assisted selection for introducing the resistance gene into common wheat.

**Table 1:** Marker name and primer sequences of polymorphic KASP markers developed from SNP markers in the genome region between the 669,955,772 and 672,333,864 bp positions on chromosome 1BL of the Chinese Spring reference genome (IWGSC Ref Seq v1.0).

Marker name	Primer sequences
A009289	Allele1 (Fam): GAAGGTGACCAAGTTCATGCTGAGCGGGAAGAGAGCAAGGGT
	Allele2 (Vic): GAAGGTCGGAGTCAACGGATTAGCGGGAAGAGAGCAAGGGG
	Com: CTTGTCCAACAGGCCCGCCAT
A009276	Allele1 (Fam): GAAGGTGACCAAGTTCATGCTCCAAAGCTGAGGGTGTCGTT
	Allele2 (Vic): GAAGGTCGGAGTCAACGGATTCCAAAGCTGAGGGTGTCGTC
	Com: AGAAGAACAAAGGCGTGATGGCGTA
A009275	Allele1 (Fam): GAAGGTGACCAAGTTCATGCTGCAAATCAATATAGCATGTTAAACAAAAAACA
	Allele2 (Vic): GAAGGTCGGAGTCAACGGATTCAAATCAATATAGCATGTTAAACAAAAAACC
	Com: GGCTATTTCTGGTTTGGCACAGGTT
A009272	Allele1 (Fam): GAAGGTGACCAAGTTCATGCTGTCGTCTCTCCAGCACACCG
	Allele2 (Vic): GAAGGTCGGAGTCAACGGATTGGTCGTCTCTCCAGCACACCA
	Com: AAGTGGAGATCATATGCTTCCATCTGAAA
A009271	Allele1 (Fam): GAAGGTGACCAAGTTCATGCTAGAGATCAAAACATACACGCAACGAAAA
	Allele2 (Vic): GAAGGTCGGAGTCAACGGATTAGAGATCAAAACATACACGCAACGAAAT
	Com: CATCCATCGCTGTATCTATATATCGTGTT
A009270	Allele1 (Fam): GAAGGTGACCAAGTTCATGCTCAATGAAATTCTCGATTTTTTAGCCGTT
	Allele2 (Vic): GAAGGTCGGAGTCAACGGATTAATGAAATTCTCGATTTTTTAGCCGTC
	Com: TAATGCACCGCAGCCATTCGACTTA
A007972	Allele1 (Fam): GAAGGTGACCAAGTTCATGCTTAAAGCCCACAACAGGCAGCG
	Allele2 (Vic): GAAGGTCGGAGTCAACGGATTAATAAAGCCCACAACAGGCAGCA
	Com: GTGTTCGTTGTCTTGTAAGACTCTAAGTT
A007975	Allele1 (Fam): GAAGGTGACCAAGTTCATGCTCGTGCTTTGCGTTCACCATATGA
	Allele2 (Vic): GAAGGTCGGAGTCAACGGATTCGTGCTTTGCGTTCACCATATGC
	Com: GCATGGCCAGGAAGAACTGTGAAAT
A007979	Allele1 (Fam): GAAGGTGACCAAGTTCATGCTAGAAATCATTGCGGTAGCCGA
	Allele2 (Vic): GAAGGTCGGAGTCAACGGATTCTAGAAATCATTGCGGTAGCCGG
	Com: GCAGTACTCCTAGCGTAACTGGTTT
A012891	Allele1 (Fam): ATAACCTAAGCTGCAGCATAACAGTA
	Allele2 (Vic): ACCTAAGCTGCAGCATAACAGTC
	Com: CAGTAAGTACTACATGCTCTGCCCTT
A012893	Allele1 (Fam): CAGGCACATGCTTAGGGATTGAG
	Allele2 (Vic): CAGGCACATGCTTAGGGATTGAC
	Com: GAACAGCGCATTTCCAGAATTTCCTAATT
A012894	Allele1 (Fam): AAGAAGTTCAAGGCATGGGCAGATA
	Allele2 (Vic): GAAGTTCAAGGCATGGGCAGATG
	Com: CTACTTCGGGAAGTACTTGTCCCAA
A012896	Allele1 (Fam): GTACGTCCACTCGCTCAAGGA
	Allele2 (Vic): GTACGTCCACTCGCTCAAGGT
	Com: CGTTATCTTTGGTGACCGCAGGATA

**Table 2:** Primer sequences were designed for the KASP markers derived from single nucleotide polymorphisms (SNPs) associated with *QYrsv.swust-1BL*, identified from the wheat 660K SNP array.

KASP ID	SNP ID	SNP physical position	SNP sequence
*A009289*	AX-111546688	669,955,772	GTGTTCGTTGTCTTGTAAGACTCTAAGTTTGTGTA[**G^a^**/T]GCTGCCTGTTGTGGGCTTTATTCAGTTAAAGCCGG
*A009276*	AX-94509279	670,180,628	ATCACTGTGGCCTCGTGCTTTGCGTTCACCATATG[**A**/C]ATTTCACAGTTCTTCCTGGCCATGCTATGCCAGAT
*A009275*	AX-109363092	670,373,377	TAGAATAATGCACCGCAGCCATTCGACTTAGATCC[A/**C**]ACGGCTAAAAAATCGAGAATTTCATTGTGTTTTTC
*A009272*	AX-109440891	670,379,326	TACTAGCAAGAGATCAAAACATACACGCAACGAAA[C/**T**]TATTAAAACAAACAAACACGATATATAGATACAGC
*A009271*	AX-109389405	670,382,321	AGTGGAGATCATATGCTTCCATCTGAAAAATAATA[A/**T**]GGTGTGCTGGAGAGACGACCTACAAACATGTAAAG
*A009270*	AX-110421026	670,475,930	AAAAGCAAATCAATATAGCATGTTAAACAAAAAAC[A/**G**]AACCTGTGCCAAACCAGAAATAGCCAAATTGGGCT
*A007979*	AX-108800083	670,502,837	ATAACCAGAAGAACAAAGGCGTGATGGCGTACACT[**T**/C]ACGACACCCTCAGCTTTGGAGATGGGCGGAACCTC
*A007975*	AX-109391895	670,524,290	ACCTGCAGTACTCCTAGCGTAACTGGTTTAGCTTT[C/**A**]CGGCTACCGCAATGATTTCTAGCGTTCCATTGTGT
*A007972*	IWB5732 (90K)	670,783,618	ACCCCTTGGCCTTGTCCAACAGGCCCGCCATGGCCG[**T**/C]CCCTTGCTCTCTTCCCGCTCCTTCCCGCCTCTGAT
*A012891*	AX-86162829	671,740,550	ACTTCTGTGGTTATAGATAGCTAAATCTATATTGT[**G**/T]ACTGTTATGCTGCAGCTTAGGTTATGATCTGTTGT
*A012893*	AX-111736421	671,741,903	CCTAATTTCATTGTTTGTGAACAGTGTCTTCCCCA[C/**G**]TCAATCCCTAAGCATGTGCCTGCGCCGGGAAACCT
*A012894*	AX-94436701	671,742,114	AATCTTTGGCTGAGAAGTTCAAGGCATGGGCAGAT[A/**G**]ACACCTCCAGGGGGTTTACCTTCTATTCGATCGGG
*A012896*	AX-94735704	672,333,864	CGTCGACCGCATCGCGTACGTCCACTCGCTCAAGG[A/**T**]GAAGCCCATCCGCATCCCCAACTATCCTGCGGTCA

**^a^** Bold indicate the nucleotide in the resistant parent Svevo.

### Multi-environment analysis of *QYrsv.swust-1BL* stripe rust resistance loci

QTL for stripe rust resistance was scanned through all 13 KASP markers by the inclusive composite interval mapping (ICIM) method implemented in Ici-Mapping V4.2 software. *QYrsv.swust-1BL* was discovered using the BIP and MET methods (LOD > 2.5, P < 0.01). *QYrsv.swust-1BL* was significant for both IT and DS, with the highest peak in a 0.72 cM region. **Figure 1** represents a summary of the analysis effects of *QYrsv.swust-1BL* using the BIP and MET models for IT and DS in multiple seasons from 2016–2020. On the BIP analysis, it has a PVE (percent of explained variation) of 10.43–24.12% in IT and a PVE of 9.32–27.34% in DS across 2016–2020 environments (**Table 3**). In the MET analysis, it has a high LOD score of 35.38 and a PVE of 11.68% in IT between markers *A007972* and *A007975*. Additionally, it has a high LOD score of 43.53 and a PVE of 24.20% in DS between markers *A009289* and *A009276* (**Table 4**).

**Table 3:** Summary of stripe rust resistance gene *QYrsv.swust-1BL* identified using the biparental population model (BIP) based on mean disease severity (DS) and infection type (IT) of the Svevo/Zavitan recombinant inbred line (RIL) population tested in China and Israel from 2016 to 2020^a^.

QTL, environment^b^	Marker	IT			DS		
		LOD	PVE	Add	LOD	PVE	Add
BD16	*A009276-A007972*	5.52	16.85	-0.82	7.02	20.91	-13.21
BK16	*A009276-A007972*	7.00	21.82	-1.01	4.75	14.92	-12.08
YL16	*A009289-A009276*	5.26	16.58	-1.09	/	/	/
QH16	*A012894-A012896* *A009276-A007972*	3.46	12.39	-0.74	3.57	12.95	-6.98
BD17	*A007972-A007975* *A009289-A009276*	7.88	24.12	-0.84	8.86	26.17	-16.93
BK17	*A007972-A007975*	3.39	10.97	-0.45	2.85	9.32	-5.99
MY17	*A009289-A009276*	5.60	17.55	-1.11	9.32	27.34	-17.39
YL17	*A009289-A009276*	5.56	17.42	-1.13	9.12	26.84	-17.85
BD18	*A007972-A007975*	3.24	10.43	-0.49	4.41	14.28	-7.50
BK18	*A009276-A007972*	4.11	13.18	-0.48	/	/	/
MY18	*A009276-A007972*	4.31	14.90	-0.81	4.91	16.97	-8.30
MY19	*A009289-A009276*	5.56	17.42	-1.13	9.12	26.84	-17.85
MY20	*A009276-A007972* *A009289-A009276*	4.11	13.18	-0.47	4.40	14.27	-7.49

^a^Marker, marker interval; LOD, logarithm of odds score; PVE, percentage of the phenotypic variance explained by individual QTL; Add, additive effect of resistance allele.^b^The Svevo/Zavitan RILs and their parents were evaluated for *QYrsv.swust-1BL* resistance to stripe rust in China fields in Yangling of Shaanxi Province in 2016 and 2017 (YL16 and YL17), Huzhu County, Qinghai Province in 2016 (QH16), and Mianyang, Sichuan Province in 2017, 2018, 2019, and 2020 (MY17, MY18, MY19, and MY20); and also in Israel fields in Bet Dagan (BD16, BD17, and BD18) and Barkai (BK16, BK17, and BK18) from 2016 to 2018.

**Table 4:** Summary of stripe rust resistance gene *QYrsv.swust-1BL* identified using the multi-environmental trials (MET) model based on the mean infection type (IT) and disease severity (DS) of the Svevo/Zavitan recombinant inbred line (RIL) population tested in China and Israel from 2016 to 2020^a^.

QTL	Pos (cM)	Left Marker	Right Marker	IT							DS						
				LOD	LOD (A)	LOD (AbyE)	PVE	PVE (A)	PVE (AbyE)	Add	LOD	LOD (A)	LOD (AbyE)	PVE	PVE (A)	PVE (AbyE)	Add
*QYrsv.swust-1BL*	0.0	*A009289*	*A009276*	26.57	19.09	7.48	20.05	8.77	11.28	-0.43	43.53	30.46	13.06	24.20	11.69	12.51	-6.98
	0.7	*A007972*	*A007975*	35.38	14.40	20.98	11.68	6.54	5.15	-0.36	31.97	16.03	15.95	10.75	6.10	4.65	-5.00

^a^Pos = the scanning position in cM on the chromosome. LOD = likelihood of odds score for all effects. LOD (A) = LOD score for additive and dominance effects. LOD (AbyE) = LOD score for additive and dominance by environment effects. PVE = phenotypic variation explained by all effects at the current scanning position. PVE (A) = phenotypic variation explained by the additive and dominance effects at the current scanning position. PVE (AbyE) = phenotypic variation explained by additive and dominance by environment effects at the current scanning position, and Add = average additive effect at the current scanning position.

### Identification of recombination events

**Figure 2:**
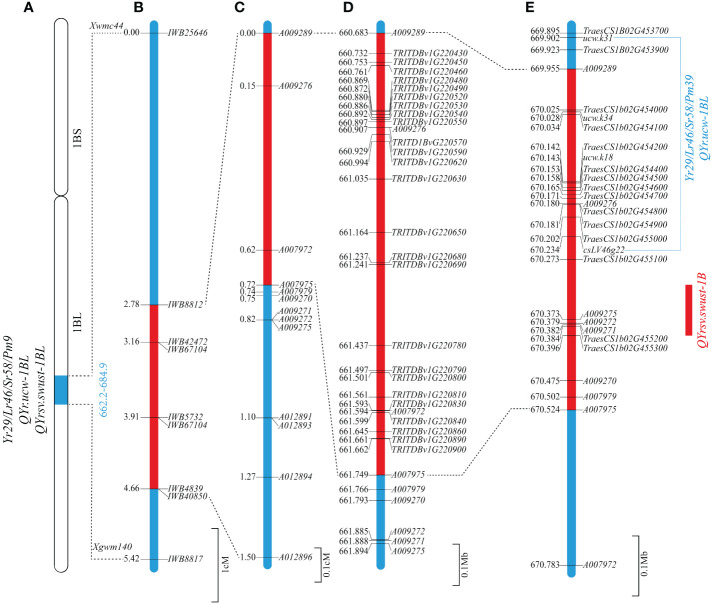
Genetic and comparative genomic linkage map of the stripe rust resistance QTL *QYrsv.swust-1BL*. **(A)** Stripe rust resistance gene/QTL: *Yr29*/*Lr46*/*Sr58*/*Pm38*, *QYr.ucw-1BL*, and *QYrsv.swust-1BL* share the same distal region in chromosome 1BL. **(B)** Genetic map constructed using a 90K SNP chip showing *QYrsv.swust-1BL* (red) within different flanked markers in comparison with *Yr29*/*Lr46*/*Sr58*/*Pm38* and *QYr.ucw-1BL* (blue). **(C)** A fine map of *QYrsv.swust-1BL* was constructed using newly developed KASP markers. **(D)** The Svevo genome region from the 660.372 Mb to 663.335 Mb position on chromosome 1B. **(E)** The Chinese Spring (RefSeq V1.0) genome region from the 669.956 Mb to 672.334 Mb position on chromosome 1B.

These 13 markers were used to genotype the 137 RILs, from which 4 RILs were found to have only one recombination event proximal to markers *A009289*, *A009276*, *A007972*, and *A007975*, respectively. Two markers (*A009289* and *A007975*) were most closely linked to the target gene on two sides and flanked the 1,065,719 bp region between the 660,683,255 bp and 661,748,974 bp positions on the durum wheat Svevo RefSeq Rel. 1.0 (**Table 2**) and the 568,518 bp region between the 669,955,772 and 670,524,290 bp positions in Chinese Spring IWGSC RefSeq v1.0 (**Table 3**) on chromosome 1BL. It is clear that *QYrsv.swust-1BL* overlaps with *QYr.ucw-1BL/Yr29*. Although the physical mapping region surpasses that of *QYr.ucw-1BL/Yr29* (669.902 ~ 670.234 Mp), it is noteworthy that only three candidate genes have been identified within the Chinese spring reference genome (**Figure 2**).

### Candidates for *QYrsv.swust-1BL*

**Figure 3:**
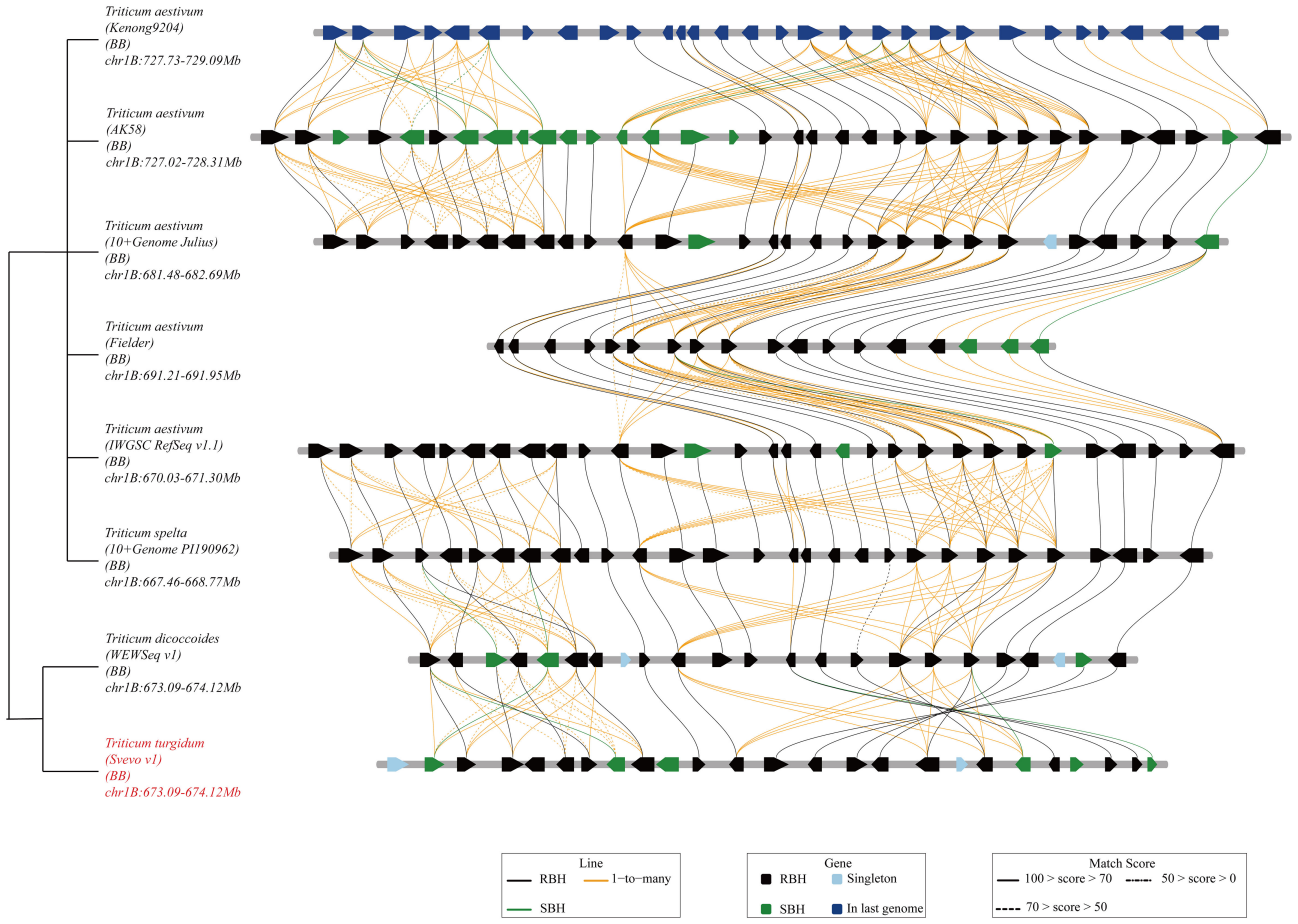
Collinearity analysis of predicted genes in *QYrsv.swust-1BL.*

Based on the analysis of BIP and MET, as well as the examination of recombination events in RILs, *QYrsv.swust-1BL* was delimited by the linked markers *A009289* and *A007975* (0.72 cM) (**Figure 2**). The genetic distance between the two flanking markers in this candidate region was found to be conservative in the durum wheat Svevo genome and 1,065,719 bp in the Svevo RefSeq Rel. 1.0 (**Figure 2**). The 1.066 Mb region was found to contain 25 annotated genes (**Figure 2**; **Table 5**). These 25 high-confidence genes in Svevo were found to be related to disease resistance. The protein encoded by five of these genes (*TRITD1Bv1G220450*, *TRITD1Bv1G220460*, *TRITD1Bv1G220520*, *TRITD1Bv1G220540*, and *TRITD1Bv1G220550*) belongs to the disease resistance protein receptor-like protein kinase, which is involved in pathogen recognition and plays an important role in effector-induced protein status monitoring (Mchale et al., 2006). Four of these genes are S-type anion channels (*TRITD1Bv1G220620*, *TRITD1Bv1G220780*, *TRITD1Bv1G220800*, and *TRITD1Bv1G220810*). Two of these genes are glucan endo-1,3-beta-glucosidase (*TRITD1Bv1G220480* and *TRITD1Bv1G220530*). The other fourteen genes are: Acid beta-fructofuranosi-dase (*TRITD1Bv1G220430*), Sugar transporter (*TRITD1Bv1G220570*), WRKY family transcription factor (*TRITD1Bv1G220590*), Band 7 stomatin family protein (*TRITD1Bv1G220630*), FAD-binding Berberine family protein (*TRITD1Bv1G220650*), Calcium-dependent protein kinase (*TRITD1Bv1G220680*), Protein ABIL1 (*TRITD1Bv1G220690*), Divalention symporter (*TRITD1Bv1G220790*), Late embryogenesis abundant protein Lea14 (*TRITD1Bv1G220830*), F-box family protein (*TRITD1Bv1G220840*), Late embryogenesis abundant hydroxyproline-rich glycoprotein family (*TRITD1Bv1G220860*), Thrombospondin type-1-domain-containing protein (*TRITD1Bv1G220890*), RING/FYVE/PHD zinc finger superfamily protein (*TRITD1Bv1G220900*), and the other is an unannotated gene (*TRITD1Bv1G220490*) (**Table 5**). There are a total of 13 candidate genes in the 568,518 bp region between positions 669,955,772 and 670,524,290 bp of the Chinese Spring IWGSC RefSeq v1.0 (**Table 5**) on chromosome 1BL. Five of the receptor-like protein kinases (*TraesCS1B02G454000*, *TraesCS1B02G454100*, *TraesCS1B02G454400*, *TraesCS1B02G454600*, and *TraesCS1B02G454700*), two of the glucan endo-1,3-beta-glucosidase (*TraesCS1B02G454200* and *TraesCS1B02G454500*), one of the sugar transporter (*TraesCS1B02G454800*), one of the WRKY family transcription factor (*TraesCS1B02G455000*), and one of the S-type anion channel (*TraesCS1B02G455100*) are candidate genes that share common genes with the Svevo genome. The other three different genes are: protein kinase (*TraesCS1B02G454900*), ATP-dependent zinc metalloprotease FtsH (*TraesCS1B02G455200*), and carboxypeptidase (*TraesCS1B02G455300*). These four classes of genes are shared between the Chinese spring genome and the Svevo genome and will be used to verify candidate genes for *QYrsv.swust-1BL* in the future. A collinearity analysis of these genes was performed using the Zavitan (*WEWSeq v1.0*), *Triticum spelta* (*spelta PI190962 v1.0)*, and other 5 *Triticum aestivum* genomes (http://wheat.cau.edu.cn/TGT/) data (**Figure 3**). The results of collinearity analysis indicate that there have been multiple chromosomal structural variations in this region. An intrachromosomal translocation exists within the range of approximately 673.1 MB to 673.8 MB, while there is a paracentric inversion within the range of 673.8 MB to 674.1 MB. Through candidate gene functional annotation. Most of the candidate genes in the Svevo reference genome have high match scores, which refer to high confidence.

**Table 5:** The putative genes identified in the genomic regions of the durum wheat Svevo genome (Ref Seq Rel. 1.0.) and the Chinese Spring genome (IWGSC v1.0) covering *QYrsv.swust-1BL*.

Svevo Reference genome			Chinese Spring Reference genome			
Gene ID	Physical positions(bp)	Predicted gene function	Gene ID	Physical positions(bp)	Predicted gene function	
*TRITD1Bv1G220430*	660,732,388	Acid beta-fructofuranosi-dase	*TraesCS1B02G454000*	670,025,361	Receptor-like protein kinase, putative, expressed	
*TRITD1Bv1G220450*	660,752,810	Receptor-like protein kinase, putative, expressed	*TraesCS1B02G454100*	670,034,244	Receptor-like protein kinase, putative, expressed	
*TRITD1Bv1G220460*	660,761,348	Receptor-like protein kinase, putative, expressed	*TraesCS1B02G454200*	670,142,373	Glucan endo-1,3-beta-glucosidase 3	
*TRITD1Bv1G220480*	660,869,208	Glucan endo-1,3-beta-glucosidase 3	*TraesCS1B02G454400*	670,152,914	Receptor-like protein kinase, putative, expressed	
*TRITD1Bv1G220490*	660,872,060	/	*TraesCS1B02G454500*	670,158,857	Glucan endo-1,3-beta-glucosidase 3	
*TRITD1Bv1G220520*	660,879,885	Receptor-like kinase	*TraesCS1B02G454600*	670,164,776	Receptor-like protein kinase, putative, expressed	
*TRITD1Bv1G220530*	660,885,534	Glucan endo-1,3-beta-glucosidase 3	*TraesCS1B02G454700*	670,170,577	Receptor-like protein kinase, putative, expressed	
*TRITD1Bv1G220540*	660,891,619	Receptor-like protein kinase, putative, expressed	*TraesCS1B02G454800*	670,180,417	Sugar transporter, putative	
*TRITD1Bv1G220550*	660,897084	Receptor-like protein kinase, putative, expressed	*TraesCS1B02G454900*	670,181,368	Protein kinase	
*TRITD1Bv1G220570*	660,906,924	Sugar transporter, putative	*TraesCS1B02G455000*	670,202,180	WRKY family transcription factor	
*TRITD1Bv1G220590*	660,928,657	WRKY family transcription factor	*TraesCS1B02G455100*	670,272,504	S-type anion channel	
*TRITD1Bv1G220620*	660,993,770	S-type anion channel	*TraesCS1B02G455200*	670,384,229	ATP-dependent zinc metalloprotease FtsH 1	
*TRITD1Bv1G220630*	661,035,030	Band 7 stomatin family protein	*TraesCS1B02G455300*	670,395,913	Carboxypeptidase	
*TRITD1Bv1G220650*	661,164,343	FAD-binding Berberine family protein				
*TRITD1Bv1G220680*	661,236,813	Calcium-dependent protein kinase				
*TRITD1Bv1G220690*	661,240,728	Protein ABIL1				
*TRITD1Bv1G220780*	661,436,635	S-type anion channel				
*TRITD1Bv1G220790*	661,497,106	Divalention symporter				
*TRITD1Bv1G220800*	661,501,096	S-type anion channel				
*TRITD1Bv1G220810*	661,560,685	S-type anion channel				
*TRITD1Bv1G220830*	661,593,365	Late embryogenesis abundant protein Lea14				
*TRITD1Bv1G220840*	661,598,766	F-box family protein				
*TRITD1Bv1G220860*	661,645,362	Late embryogenesis abundant hydroxyproline-rich glycoprotein family, putative				
*TRITD1Bv1G220890*	661,660,533	Thrombospondin type-1-domain-containing protein 7B				
*TRITD1Bv1G220900*	661,662,345	RING/FYVE/PHD zinc finger superfamily protein TE				

### *QYrsv.swust-1BL* KASP marker validation

**Figure 4:**
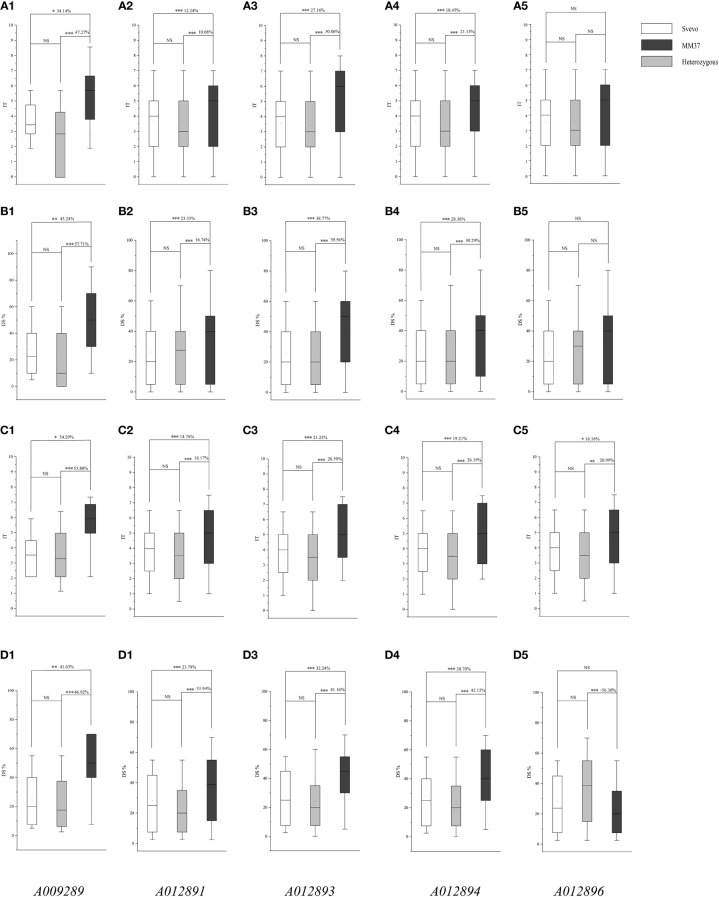
Effects of *QYrsv.swust-1BL* in the F_2_ and F_2:3_ validation populations. The lines with *QYrsv.swust-1BL* had lower IT and DS than the lines carrying the allele gene of MM37, and the difference between Svevo and heterozygous is not significant. **(A1-A5, B1-B5)** are based on the IT and DS data of the F2 population, and **(C1-C5, D1-D5)** are based on the IT and DS data of the F_2:3_ lines. Numbers 1-5 refer to the validation by five KASP markers *A009289, A012891, A012893, A012894* and *A012896*. * Significance level at P < 0.05; ** Significance level at P <0.01; *** Significance level at P < 0.005. “ns” refers to the none significance.

To assess the applicability of the newly developed KASP marker in breeding programs, an F_2_ breeding population derived from a cross between the Chinese hexaploid wheat variety Mianmai 37 and the durum wheat variety Svevo was utilized. The developed KASP markers *A009289*, *A012891*, *A012893*, *A012894*, and *A012896* effectively distinguished genotypes within the F_2_ breeding population into three distinct clusters (**Figure 4**). The allele cluster nucleotide of homozygote associated with resistance for KASP markers *A009289*, *A012891*, *A012893*, *A012894*, and *A012896* were identified as ‘G’, ‘G’, ‘G’, ‘G’, ‘T’, respectively, while the alternative allele had a nucleotide cluster of ‘T’, ‘T’, ‘C’, ‘A’, ‘A’, respectively (**Table 2**; **Figure 4**). F_2_ plants and corresponds to 318 F_2:3_ lines, which were phenotyped for stripe rust response. Among homozygous alleles associated with resistance for marker *A009289*, F_2_ plants carrying positive alleles from *QYrsv.swust-1BL* exhibited a reduction in IT and DS by 34.14% and 45.24% compared with those of the alternative allele ‘T’, respectively. Progenies of F_2:3_ carrying positive alleles from *QYrsv.swust-1BL* exhibited a reduction in IT and DS by 54.29% and 41.63% compared with those of the alternative allele ‘T’, respectively. Progenies with heterozygous genotypes for marker *A009289* also demonstrated a decrease in IT and DS by 47.27 and 57.71 in F_2_ plants and 53.80% and 46.92% in F_2:3_ lines compared with those of the alternative allele ‘T’, respectively. Conversely, progenies carrying alternative alleles from Mianmai 37 showed an increase in IT and DS. The phenotypes of F_2_ plants and F_2:3_ families carrying positive alleles and heterozygous genotypes with *A012891*, *A012893*, and *A012894* markers were significantly different from those carrying alternative alleles, with a difference of 0.005 level, similar to the results of *the A009289* marker. The results of marker *A012896* in F_2_ generation were not significant in IT and DS, while the IT differences in F_2:3_ generation were significant at the levels of 0.05 and 0.01. These findings highlight the effective utilization of KASP markers *A009289*, *A012891*, *A012893*, *A012894*, and *A012896* for marker-assisted selection in breeding programs, confirming that *QYrsv.swust-1BL* is indeed a significant and stable major gene locus controlling resistance to stripe rust disease.

## Discussion

Conventional phenotypic screening approaches have been used to develop cultivars with long-lasting disease resistance, but the breeding process takes many years. Molecular markers associated with rust resistance can be used to speed up the process and stack different resistance genes into a single cultivar. In a previous study, a QTL, *QYrsv.swust-1BL*, for APR to stripe rust was identified and mapped in chromosome 1BL in durum wheat Svevo (Zhou et al., 2021). By comparing the genetic and physical positions of linked molecular markers, *QYrsv.swust-1BL* was found to be in the same position as *Yr29* on chromosome 1BL.

In the present study, we used the high-density wheat 660K SNP array to find more SNP markers in the *QYrsv.swust-1BL* region and successfully converted the SNP markers into KASP markers. By re-mapping *QYrsv.swust-1BL* using the same Svevo/Zavitan RIL population as used in the previous study (Zhou et al., 2021), we added 13 KASP markers to the target region with a much higher resolution. These KASP markers were validated using an F_2_ population of 318 plants from a cross of Svevo with the common elite wheat cultivar MM 37. The genetic distance of the *QYrsv.swust-1BL* interval was reduced to 0.72 cM (**Figure 2**). Finally, we mapped this stripe rust resistance locus within a 1.066 Mb region of chromosome 1BL in durum wheat Svevo RefSeq Rel. 1.0, which overlaps with the previously reported genes, *Yr29/Lr46/Sr58/Pm39*, for resistance to stripe rust (yellow rust), leaf rust, stem rust, and powdery mildew, respectively (William et al., 2003; Lillemo et al., 2008; Singh et al., 2013). The genomic region between markers *A009289* and *A007975* in the Chinese Spring reference genome was found to be 0.569 Mb, comparable to the size of the region in the durum wheat genome.

Based on the results of high-resolution mapping using a large population, exome capture, and allelism analysis of a cross between common wheat RIL55 (with the Klein Chajá allele of *QYr.ucw-1BL*) and Lalbahadur (with the Pavon-1B allele of *Yr29*), Cobo et al. (2019) reported that *QYr.ucw-1BL* and *Yr29* represent the same gene. According to the comparative maps and annotated genes, we hypothesized that *QYrsv.swust-1BL* should be the same as *QYr.ucw-1BL/Yr29*. The genomic interval between KASP markers A009289 and A007975 in the Chinese Spring genome (IWGSC v1.0) spans a smaller region of 0.569 Mb and shares four complete classes of genes. However, the 1.066 Mb region between KASP markers *A009289* (at the 660,683,255 bp position) and *A007975* (at the 661,748,974 bp position) contains 25 high-confidence genes annotated in the Svevo reference genome, including the *QYr.ucw-1BL* region (Cobo et al., 2018, 2019). These 25 and 13 genes in Svevo RefSeq Rel. 1.0 and Chinese Spring IWGSC RefSeq v1.0 are listed in **Table 5**, respectively. Ten annotated genes were reported in the *QYr.ucw-1BL* region between markers *ucw.k31* and *csLV46G22* (Cobo et al., 2019). As *QYrsv.swust*-*1BL* and *QYr.ucw-1BL/Yr29* were mapped to the same regions of the Chinese Spring genome, we found four classes of identical genes in these genomic regions. Thus, *QYrsv.swust*-*1BL* and *QYr.ucw-1BL/Yr29* are likely the same gene. More conclusive evidence for these two QTL as the same gene needs future studies of direct comparison of both QTL in common wheat backgrounds through allelism testing, gene cloning, and expression. The F_2:3_ lines or later generation lines with 42 chromosomes from the MM 37/Svevo cross can be used together with the *QYrsv.swust*-*1BL/QYr.ucw-1BL/Yr29* near-isogenic lines in the Avocet S background for such experiments.

In the present study, we showed that the common wheat Chinese Spring and durum wheat Svevo genome sequences can be used to hasten high-resolution mapping of traits of interest in wheat. We encountered difficulties, such as sterile plants caused by aneuploids from the common wheat and durum wheat crosses, when attempting to clone *QYrsv.swust*-*1BL* because of the ploid differences between the parental lines and the reference genomes. Therefore, regions like *QYrsv.swust*-*1BL* from durum wheat in this study should be assembled using the donor genome to enable the cloning of causal genes and to develop diagnostic markers that can be used in breeding programs.

For breeding wheat cultivars with stripe rust resistance, *QYrsv.swust*-*1BL* can be used together with other effective resistance genes. The progeny lines with 42 chromosomes and *QYrsv.swust*-*1BL* derived from cross MM 37/Svevo should be more efficient than the original donor of durum wheat for incorporating the APR QTL into other common wheat backgrounds. The KASP markers tightly linked to *QYrsv.swust*-*1BL* can be used in marker-assisted selection for speeding up its incorporation and pyramiding with other effective genes for developing wheat cultivars with high-level, durable resistance to stripe rust.

## Conclusion

In this study, using high-density 660K SNP array genotyping, we fine-mapped the *QYrsv.swust-1BL* APR locus as a starting point to develop a diagnostic marker for use in breeding and to clone this gene. We mapped *QYrsv.swust-1BL* to within a 1.066 Mb region in *Triticum turgidum* durum wheat Svevo (RefSeq Rel. 1.0) on the chromosome arm 1BL, which overlaps with a previously described map of *QYr.ucw-1BL*/*Yr29*, a *Pst* resistance gene. The four gene families within the identified 1.066 Mb region, identical to the Chinese spring reference genome, have been implicated in disease resistance. SNP markers were then used to select high-throughput KASP markers that could be used in wheat breeding programs to hasten the deployment of these *Pst* resistance loci.

## Data availability statement

The original contributions presented in the study are included in the article/supplementary material. Further inquiries can be directed to the corresponding authors.

## Author contributions

XZ: Writing – review & editing, Writing – original draft, Visualization, Investigation, Formal analysis, Data curation. GJ: Writing – review & editing, Writing – original draft, Visualization, Investigation, Formal analysis, Data curation. YL: Writing – review & editing. XL: Writing – review & editing, Supervision. LC: Writing – review & editing, Visualization, Supervision, Data curation. XC: Writing – review & editing, Visualization, Supervision, Investigation, Data curation. ZK: Writing – review & editing, Visualization, Supervision.

## References

[B1] Anderson J. A. Churchill G. A. Autrique J. E. Tanksley S. D. Sorrells M. E. (1993). Optimizing parental selection for genetic-linkage maps. Genome 36, 181–186. doi: 10.1139/g93-024 18469981

[B2] Avni R. Nave M. Eilam T. Sela H. Alekperov C. Peleg Z.. (2014). Ultra-dense genetic map of durum wheat × wild emmer wheat developed using the 90K iSelect SNP genotyping assay. Mol. Breed. 34, 1549–1562. doi: 10.1007/s11032-014-0176-2

[B3] Bansal U. K. Kazi A. G. Singh B. Hare R. A. Basiana H. S. (2014). Mapping of durable stripe rust resistance in a durum wheat cultivar Wollaroi. Mol. Breed. 33, 51–59. doi: 10.1007/s11032-013-9933-x

[B4] Beddow J. M. Pardey P. G. Chai Y. Hurley T. M. Kriticos D. J. Braun H. J.. (2015). Research investment implications of shifts in the global geography of wheat stripe rust. Nat. Plants 1, 1–5. doi: 10.1038/nplants.2015.132 27251389

[B5] Chen X. M. (2005). Epidemiology and control of stripe rust (*Puccinia striiformis* f. sp. *tritici*) on wheat. Can. J. Plant Pathol. 27, 314–337. doi: 10.1080/07060660509507230

[B6] Chen X. M. Coram T. Huang X. L. Wang M. N. Dolezal A. (2013). Understanding molecular mechanisms of durable and non-durable resistance to stripe rust in wheat using a transcriptomics approach. Cur. Genomics 14, 111–126. doi: 10.2174/1389202911314020004 PMC3637676 24082821

[B7] Chen W. Q. Wellings C. Chen X. M. Kang Z. S. Liu T. G. (2014). Wheat stripe (yellow) rust caused by *Puccinia striiformis* f. sp. *tritici* . Mol. Plant Pathol. 15, 433–446. doi: 10.1111/mpp.12116 24373199 PMC6638732

[B8] Chen J. Upadhyaya N. M. Ortiz D. Sperschneider J. Li F. Bouton C.. (2017). Loss of AvrSr50 by somatic exchange in stem rust leads to virulence for *Sr50* resistance in wheat. Science 358, 1607–1610. doi: 10.1126/science.aao4810 29269475

[B9] Chen S. Zhang W. Bolus S. Rouse M. N. Dubcovsky J. (2018). Identification and characterization of wheat stem rust resistance gene *Sr21* effective against the Ug99 race group at high temperature. PloS Genet. 14, e1007287. doi: 10.1371/journal.pgen.1007287 29614079 PMC5882135

[B10] Chen X. M. (2020). Pathogens which threaten food security: *Puccinia striiformis*, the wheat stripe rust pathogen. Food Secur. 12, 239–251. doi: 10.1007/s12571-020-01016-z

[B11] Cobo N. Pflüger L. Chen X. M. Dubcovsky J. (2018). Mapping QTL for resistance to new virulent races of wheat stripe rust from two argentinean wheat cultivars. Crop Sci. 58, 2470–2483. doi: 10.2135/cropsci2018.04.0286

[B12] Cobo N. Wanjugi H. Lagudah E. Dubcovsky J. (2019). A high-resolution map of wheat *QYr.ucw-1BL*, an adult plant stripe rust resistance locus in the same chromosomal region as *Yr29* . Plant Genome 12, 180055. doi: 10.3835/plantgenome2018.08.0055 PMC12962344 30951084

[B13] Feng J. Y. Wang M. N. See D. R. Chao S. M. Zheng Y. L. Chen X. M. (2018). Characterization of novel gene *Yr79* and four additional QTL for all-stage and high-temperature adult-plant resistance to stripe rust in spring wheat PI 182103. Phytopathology 108, 737–747. doi: 10.1094/PHYTO-11-17-0375-R 29303685

[B14] Feng J. Yao F. Wang M. See D. R. Chen X. (2023). Molecular mapping of *Yr85* and comparison with other genes for resistance to stripe rust on wheat chromosome 1B. Plant Dis. 0, null. doi: 10.1094/PDIS-11-22-2600-RE 37221244

[B15] Fu D. Uauy C. Distelfeld A. Blechl A. Epstein L. Chen X. M.. (2009). A kinase-START gene confers temperature-dependent resistance to wheat stripe rust. Science 323, 1357–1360. doi: 10.1126/science.1166289 19228999 PMC4737487

[B16] Gessese M. Bariana H. Wong D. Hayden M. Bansal U. (2019). Molecular mapping of stripe rust resistance gene *Yr81* in a common wheat landrace Aus27430. Plant Dis. 103, 1166–1171. doi: 10.1094/PDIS-06-18-1055-RE 30998448

[B17] Gou J. Y. Li K. Wu K. Wang X. Lin H. Cantu D.. (2015). Wheat stripe rust resistance protein WKS1 reduces the ability of the thylakoid-associated ascorbate peroxidase to detoxify reactive oxygen species. Plant Cell 27, 1755–1770. doi: 10.1105/tpc.114.134296 25991734 PMC4498197

[B18] Herrera-Foessel S. A. Singh R. P. Huerta-Espino J. Salazar V. C. Lagudah E. S. (2011). “First report of slow rusting gene *Lr46* in durum wheat,” in Poster Abstracts of the Borlaug Global Rust Initiative Technical Workshop. Ed. McIntosh M. (Saint Paul, Minnesota, USA), 191.

[B19] Herrera-Foessel S. A. Singh R. P. Lan C. X. Huerta-Espino J. Calvo-Salazar V. Bansal U. K.. (2015). *Yr60*, a gene conferring moderate resistance to stripe rust in wheat. Plant Dis. 99, 508–511. doi: 10.1094/PDIS-08-14-0796-RE 30699549

[B20] Klymiuk V. Chawla H. S. Wiebe K. Ens J. Fatiukha A. Govta L.. (2022). Discovery of stripe rust resistance with incomplete dominance in wild emmer wheatusing bulked segregant analysis sequencing. Commun. Biol. 5, 826. doi: 10.1038/s42003-022-03773-3 35978056 PMC9386016

[B21] Kolmer J. A. (2015). A QTL on chromosome 5BL in wheat enhances leaf rust resistance of *Lr46* . Mol. Breed. 35, 74–81. doi: 10.1007/s11032-015-0274-9

[B22] Krattinger S. G. Lagudah E. S. Spielmeyer W. Singh R. P. Huerta-Espino J. McFadden H.. (2009). A putative ABC transporter confers durable resistance to multiple fungal pathogens in wheat. Science 323, 1360–1363. doi: 10.1126/science.1166453 19229000

[B23] Li J. Dundas I. Dong C. Li G. Trethowan R. Yang Z.. (2020). Identification and characterization of a new stripe rust resistance gene *Yr83* on rye chromosome 6R in wheat. Theor. Appl. Genet. 133, 1095–1107. doi: 10.1007/s00122-020-03534-y 31955232

[B24] Lillemo M. Asalf B. Singh R. P. Huerta-Espino J. Chen X. M. He Z. H.. (2008). The adult plant rust resistance loci *Lr34/Yr18* and *Lr46/Yr29* are important determinants of partial resistance to powdery mildew in bread wheat line Saar. Theor. Appl. Genet. 116, 1155–1166. doi: 10.1007/s00122-008-0743-1 18347772

[B25] Lillemo M. Joshi A. K. Prasad R. Chand R. Singh R. P. (2013). QTL for spot blotch resistance in bread wheat line Saar co-locateto the biotrophic disease resistance loci *Lr34* and *Lr46* . Theor. Appl. Genet. 126, 711–719. doi: 10.1007/s00122-012-2012-6 23139144

[B26] Line R. F. Qayoum A. (1992). Virulence, aggressiveness, evolution and distribution of races of *Puccinia striiformis* (the cause of stripe rust of wheat) in North America 1968–1987 (Washington, DC: US Department of Agriculture).

[B27] Liu L. Wang M. N. Feng J. Y. See D. R. Chao S. M. Chen X. M. (2018). Combination of all-stage and high-temperature adult-plant resistance QTL confers high level, durable resistance to stripe rust in winter wheat cultivar Madsen. Theor. Appl. Genet. 131, 1835–1849. doi: 10.1007/s00122-018-3116-4 29797034

[B28] Liu L. Yuan C. Y. Wang M. N. See D. R. Zemetra R. S. Chen X. M. (2019). QTL analysis of durable stripe rust resistance in the North American winter wheat cultivar Skiles. Theor. Appl. Genet. 132, 1677–1691. doi: 10.1007/s00122-019-03307-2 30796480

[B29] Maccaferri M. Harris N. S. Twardziok S. O. Pasam R. K. Gundlach H. Spannagl M.. (2019). Durum wheat genome highlights past domestication signatures and future improvement targets. Nat. Genet. 51, 885–895. doi: 10.1038/s41588-019-0381-3 30962619

[B30] Marchal C. Zhang J. Zhang P. Fenwick P. Steuernagel B. Adamski N. M.. (2018). BED-domain containing immune receptors confer diverse resistance spectra to yellow rust. Nat. Plants 4, 662–668. doi: 10.1038/s41477-018-0236-4 30150615

[B31] Mchale L. Tan X. Koehl P. Michelmore R. W. (2006). Plant NBS-LRR proteins: adaptable guards. Genome Biol. 7, 212. doi: 10.1186/gb-2006-7-4-212 16677430 PMC1557992

[B32] Meng L. Li H. H. Zhang L. Y. Wang J. K. (2015). QTL IciMapping: integrated software for genetic linkage map construction and quantitative trait locus mapping in biparental populations. Crop J. 3, 269–283. doi: 10.1016/j.cj.2015.01.001

[B33] Moore J. W. Herrera-Foessel S. Lan C. Schnippenkoetter W. Ayliffe M. Huerta-Espino J.. (2015). A recently evolved hexose transporter variant confers resistance to multiple pathogens in wheat. Nat. Genet. 47, 1494–1498. doi: 10.1038/ng.3439 26551671

[B34] Nsabiyera V. Bariana H. S. Qureshi N. Wong D. Hayden M. J. Bansal U. K. (2018). Characterization and mapping of adult plant stripe rust resistance in wheat accession Aus27284. Theor. Appl. Genet. 131, 1–9. doi: 10.1007/s00122-018-3090-x 29560515

[B35] Pakeerathan K. Bariana H. Qureshi N. Wong D. Hayden M. Bansal U. (2019). Identification of a new source of stripe rust resistance *Yr82* in wheat. Theor. Appl. Genet. 132, 3169–3176. doi: 10.1007/s00122-019-03416-y 31463519

[B36] Periyannan S. Moore J. Ayliffe M. Bansal U. Wang X. Huang L.. (2013). The gene *Sr33*, an ortholog of barley *Mla* genes, encodes resistance to wheat stem rust race Ug99. Science 341, 786–788. doi: 10.1126/science.1239028 23811228

[B37] Ponce-Molina L. J. Huerta-Espino J. Singh R. Basnet B. R. Lagudah E. S. Aguilar-Rincón V. H.. (2018). Characterization of adult plant resistance to leaf rust and stripe rust in Indian wheat cultivar ‘New Pusa 876’. Crop Sci. 58, 630–638. doi: 10.2135/cropsci2017.06.0396

[B38] Ramirez-Gonzalez R. H. Uauy C. Caccamo M. (2015). PolyMarker: a fast polyploid primer design pipeline. Bioinformatics 31, 2038–2039. doi: 10.1093/bioinformatics/btv069 25649618 PMC4765872

[B39] Ren Y. Singh R. P. Basnet B. R. Lan C. X. Huerta-Espino J. Lagudah E. S.. (2017). Identification and mapping of adult plant resistance loci to leaf rust and stripe rust in common wheat cultivar Kundan. Plant Dis. 101, 456–463. doi: 10.1094/PDIS-06-16-0890-RE 30677352

[B40] Rosewarne G. M. Singh R. P. Huerta-Espino J. William H. M. Bouchet S. Cloutier S.. (2006). Leaf tip necrosis, molecular markers and β1-proteasome subunits associated with the slow rusting resistance genes *Lr46/Yr29* . Theor. Appl. Genet. 112, 500–508. doi: 10.1007/s00122-005-0153-6 16331478

[B41] Salcedo A. Rutter W. Wang S. Akhunova A. Bolus S. Chao S.. (2017). Variation in the *AvrSr35* gene determines *Sr35* resistance against wheat stem rust race Ug99. Science 358, 1604–1606. doi: 10.1126/science.aao7294 29269474 PMC6518949

[B42] Singh R. P. Herrera-Foessel S. A. Huerta-Espino J. Lan C. X. Basnet B. R. Bhavani S.. (2013). “Pleiotropic gene *Lr46/Yr29/Pm39/Ltn2* confers slow rusting, adult plant resistance to wheat stem rust fungus,” in Proceedings of the Borlaug Global Rust Initiative Technical Workshop, 19–22 Aug (Indian Council of Agricultural Research, New Delhi), 17.1.

[B43] Singh R. P. Huerta-Espino J. Bhavani S. Herrera-Foessel S. A. Singh D. Singh P. K.. (2011). Race non-specific resistance to rust diseases in CIMMYT spring wheats. Euphytica 179, 175–186. doi: 10.1007/s10681-010-0322-9

[B44] Singh R. P. Mujeeb-Kazi A. Huerta-Espino J. (1998). *Lr46*: A gene conferring slow-rusting resistance to leaf rust in wheat. Phytopathology 88, 890–894. doi: 10.1094/PHYTO.1998.88.9.890 18944865

[B45] Singh A. K. Zhang P. Dong C. Li J. Singh S. Trethowan R.. (2021). Generation and molecular marker and cytological characterization of wheat - Secale strictum subsp. anatolicum derivatives. Genome 64, 29–38. doi: 10.1139/gen-2020-0060 33002386

[B46] Wang J. K. (2009). Inclusive composite interval mapping of quantitative trait genes. Acta Phytopathol. Sin. 35, 239–245. doi: 10.3724/SP.J.1006.2009.00239

[B47] Wang M. N. Chen X. M. (2017). “Stripe rust resistance,” in Stripe Rust. Eds. Chen X. M. Kang Z. S. (Springer, Dordrecht), 353–558. doi: 10.1007/978-94-024-1111-9

[B48] William H. M. Singh R. P. Huerta-Espino J. Islas S. O. Hoisington D. (2003). Molecular marker mapping of leaf rust resistance gene *Lr46* and its association with stripe rust resistance gene *Yr29* in wheat. Phytopathology 93, 153–159. doi: 10.1094/PHYTO.2003.93.2.153 18943129

[B49] Wu J. H. Zeng Q. D. Wang Q. L. Liu S. J. Yu S. Z. Mu J. M.. (2018). SNP−based pool genotyping and haplotype analysis accelerate fine−mapping of the wheat genomic region containing stripe rust resistance gene *Yr26* . Theor. Appl. Genet. 131, 1481–1496. doi: 10.1007/s00122-018-3092-8 29666883

[B50] Yao E. Blake V. C. Cooper L. Wight C. P. Michel S. Cagirici H. B. (2022). “Graingenes: a data-rich repository for small grains genetics and genomics”. Database, 2022, baac034. doi: 10.1093/database/baac034 35616118 PMC9216595

[B51] Zadoks J. C. Chang T. T. Konzak C. F. (1974). A decimal code for the growth stages of cereals. Weed Res. 14, 415–421. doi: 10.1111/j.1365-3180.1974.tb01084.x

[B52] Zhang W. Chen S. Abate Z. Nirmala J. Rouse M. N. Dubcovsky J. (2017). Identification and characterization of *Sr13*, a tetraploid wheat gene that confers resistance to the Ug99 stem rust race group. Proc. Natl. Acad. Sci. U.S.A. 114, E9483–E9492. doi: 10.1073/pnas.1706277114 29078294 PMC5692537

[B53] Zhou X. L. Hu T. Li X. Yu M. Li Y. Yang S.. (2019). Genome-wide mapping of adult plant stripe rust resistance in wheat cultivar Toni. Theor. Appl. Genet. 132, 1693–1704. doi: 10.1007/s00122-019-03308-1 30941466

[B54] Zhou X. L. Zhong X. Roter J. Li X. Yao Q. Yan J. H.. (2021). Genome-wide mapping of adult plant stripe rust resistance locus derived from durum wheat Svevo by 90K SNP array. Plant Dis. 105, 879–888. doi: 10.1094/PDIS-09-20-1933-RE 33141640

[B55] Zhu Z. Cao Q. Han D. Wu J. Wu L. Tong J.. (2023). Molecular characterization and validation of adult-plant stripe rust resistance gene *Yr86* in Chinese wheat cultivar Zhongmai 895. Theor. Appl. Genet. 136, 142. doi: 10.1007/s00122-023-04374-2 37247049

